# Comment on Brinkis et al. Nutrient Intake with Early Progressive Enteral Feeding and Growth of Very Low-Birth-Weight Newborns. *Nutrients* 2022, *14*, 1181

**DOI:** 10.3390/nu14132651

**Published:** 2022-06-27

**Authors:** Shabih Manzar

**Affiliations:** Department of Pediatrics, Louisiana State University Health Center, Shreveport, LA 71103, USA; shabih.manzar@lsuhs.edu; Tel.: +1-318-626-1623; Fax: +1-318-675-6059

I read with great interest the article by Brinkis et al. [1] demonstrating that early progressive enteral feeding with human milk was well tolerated in very low birth weight (VLBW) infants and concluding that target enteral nutrient intake may be reached early and improve in-hospital growth. They reported 14% (6 cases) of combined necrotizing enterocolitis (NEC) and spontaneous intestinal perforation (SIP) among group 1, <1000 g, in Table 1 [1]. They excluded three infants who had partial bowel removal from the study, as shown in Figure 1 [1]. However, Table 1 [1] showed a total count of 120: 43 in group 1, plus 43 in group 2, plus 34 in group 3. If we add these three excluded cases to the six other cases of NEC/SIP in group 1, the total would be nine, corresponding to 34% (9/43), assuming that all the bowel resection cases were in group 1 (<1000 g). Further explanation is required to alleviate this source of potential confusion.

Looking at Figure 3e [1], which displays the total fluid intake in mL/kg/day, the median day 7 parental fluid was 65 mL/kg/day, while median day 7 enteral fluid was 140 m/kg/day, giving an impression of high per day fluid use. In text, the authors mentioned using fluid up to 180 mL/kg/day; interestingly, there was no mention of incidence of patent ductus arteriosus (PDA) in the study cohort. In a recent study, Mirza et al. [2] showed higher fluid intake in the first 2 days of life in preterm infants to be associated with prolonged duration of PDA.

The authors did acknowledge that there is high variability in defining the optimal postnatal growth of very preterm infants. Some researchers use standard deviation, some use z-score, while others use growth velocity. We recently suggested using a triple method of growth assessment in preterm infants [3], combining growth velocity (GV), weight gain ratio (WGR), and delta z-score (ΔZ). The triple method was derived from the reports of Patel et al. and Rochow et al. [4,5]. A GV of 10–15 g/kg/d, a WGR near 1, and a ΔZ close to 0 was considered to be reassuring (Table 1).

In conclusion, early progressive enteral feeding is essential in VLBW infants; however, more data on the incidence of NEC, SIP, and PDA are needed, especially in infants <1000 g.

## Figures and Tables

**Table 1:** Growth Monitoring Formulae [4,5].

Method	Formula	Expected Values
Growth velocity (GV)(g/kg/d)	Current weight—Previous weight/Average weight × 1000/number of days	10–15 g/kg/d
Weight Gain Ratio (WGR)	Current weight—Previous weight/50% Weight difference	1
Z-score	Current Z score—Previous Z score	Close to 0
